# Transcriptome profiling reveals regulatory mechanisms underlying corolla senescence in petunia

**DOI:** 10.1038/s41438-018-0018-1

**Published:** 2018-04-01

**Authors:** Hong Wang, XiaoXiao Chang, Jing Lin, Youhong Chang, Jen-Chih Chen, Michael S. Reid, Cai-Zhong Jiang

**Affiliations:** 1 Institute of Pomology/Jiangsu Key Laboratory for Horticultural Crop Genetic Improvement, Jiangsu Academy of Agricultural Sciences, 210014 Nanjing, China; 2 Department of Plant Sciences, University of California, Davis, Davis, CA 95616 USA; 3 Institute of Fruit Tree Research, Guangdong Academy of Agricultural Science, 510640 Guangzhou, China; 4 Institute of Biotechnology, National Taiwan University, 10617 Taipei, Taiwan; 5 United States Department of Agriculture, Crops Pathology and Genetics Research Unit, Agricultural Research Service, Davis, CA 95616 USA

## Abstract

The genetic regulatory mechanisms that govern natural corolla senescence in petunia are not well understood. To identify key genes and pathways that regulate the process, we performed a transcriptome analysis in petunia corolla at four developmental stages, including corolla fully opening without anther dehiscence (D0), corolla expansion, 2 days after anthesis (D2), corolla with initial signs of senescence (D4), and wilting corolla (D7). We identified large numbers of differentially expressed genes (DEGs), ranging from 4626 between the transition from D0 and D2, 1116 between D2 and D4, a transition to the onset of flower senescence, and 327 between D4 and D7, a developmental stage representing flower senescence. KEGG analysis showed that the auxin- and ethylene-related hormone biosynthesis and signaling transduction pathways were significantly activated during the flower development and highly upregulated at onset of flower senescence. Ethylene emission was detected at the D2 to D4 transition, followed by a large eruption at the D4 to D7 transition. Furthermore, large numbers of transcription factors (TFs) were activated over the course of senescence. Functional analysis by virus-induced gene silencing (VIGS) experiments demonstrated that inhibition of the expression of TFs, such as ethylene-related ERF, auxin-related ARF, bHLH, HB, and MADS-box, significantly extended or shortened flower longevity. Our data suggest that hormonal interaction between auxin and ethylene may play critical regulatory roles in the onset of natural corolla senescence in petunia.

## Flower development: When to wilt

How long flowers bloom is determined by genes that alter levels of the plant hormones ethylene and auxin. When flowers are no longer needed, plants signal them to die; the underlying genetic mechanisms are not well understood. Cai-Zhong Jiang at the University of California, Davis and co-workers used genetic analysis to determine how the signals for petal death are encoded. They studied petunia flowers at four stages: opening, pollen release, onset of wilting, and fully wilted, and identified over 5000 genes that were turned up or down. The master switches for petal death were found to be genes controlling ethylene and auxin levels. Using a virus to artificially switch off several genes, the researchers identified genes that shorten or extend flower life by up to three days. These results may be useful in plant breeding.

## Introduction

Petal senescence is the irreversible and final stage of floral differentiation and development, associated with dynamic alterations once a flower has been successfully pollinated^1,2^. However, it is not clear how the process is regulated genetically. Homeostasis or alterations of plant hormones is involved in the onset of floral senescence^3^. In ethylene (ET)-sensitive flowers, the first sign of visible senescence is accompanied by a transient and sudden rise of ET production^4^. Other hormones such as cytokinin (CK)^5^, abscisic acid (ABA), auxin^6^, gibberellic acid (GA)^7^, and jasmonic acid (JA)^8^ are also involved in ET-sensitive petal senescence. ABA accelerates petal senescence^9^. Treatment with ABA promotes the large increase in ET production and hastens petal wilting in carnation flowers^10^. Pretreatments with silver thiosulfate (STS), a chemical that inhibits the perception of ET by the ET receptor, completely prevents the increase in ABA levels^11^. A negative relationship was observed between the level of CKs and petal senescence in petunia and carnation. In rose, the increase of CK content antagonized petal senescence prompted by ET^12^. Applying CKs delayed petunia petal senescence^13^. Auxin also plays a role in ET-sensitive petal senescence. Application of auxin prompted ET production and petal wilting in cut carnation flowers^14^. In addition, 2,4-dichlorophenoxyacetic acid (2,4-d), a synthetic auxin, induced the expression of 1-aminocyclopropane-1-carboxylic acid synthase (*ACS*) genes in petals^15^. In most research, these hormones are used as exogenous regulators to observe ET sensitivity and floral longevity in ET-sensitive species. Although the enrichment of ‘response to 1-aminocyclopropase-1-carboxylic acid (ACC) and auxin stimulus’ was observed 12 h after pollination in the petals of petunia^16^, the differential expression patterns of genes related to these hormones in petal senescence is unclear.

Petal senescence is regulated by transcription factors (TFs). On one hand, ethylene-insensitve-like (EIL) and ethylene response factors (ERFs) are correlated with the ET response signaling pathway^2^. *EIL3*, a homolog of ET-insensitive 3 (EIN3) in carnation, is a pivotal switch of ET-induced gene expression^6,17^. *DAFSAG9*, which is homologous to *ERF2*, was significantly upregulated in senescing daffodil petals^18^. On the other hand, a large group of other TFs, such as B-box zinc finger, bHLH DNA-binding, homeodomain-like (HD), MADS-box, MYB, and NAC, display differential expression when ET-insensitivity is induced in the *etr1-1* transgenic petunia^3^. More than 20 members from the ERF, NAC, bZIP, HD-Zip, and WRKY TF families showed differential expression in petals at the early stage of pollination-induced senescence in petunia^16^. In addition, NAC, Aux/IAA, MYB, bZIP, and MADS-box are differentially expressed during carnation petal senescence^6,19^. These studies indicate that these TFs play regulating roles in ET-dependent petal senescence. However, the biological functions of these TFs are largely unknown.

High-throughput gene expression analysis using messenger RNA (mRNA) sequencing (RNA-Seq) represents the most powerful tool to elucidate the underlying regulatory mechanism of corolla senescence. Recently, pollination- and ET-induced corolla senescence in petunia has been studied through RNA-Seq analysis^16,20^, however, the regulatory mechanisms that govern the onset of natural corolla senescence from opening to wilting in petunia is unclear. Therefore, identifying the dynamic processes and regulatory factors in transcription is a crucial step in determining the master switches in corolla senescence. We employed RNA-seq technology to investigate the global and chronological sequence of transcriptional events during the initial corolla senescence in petunia. Furthermore, virus-induced gene silencing (VIGS) system was used to dissect biological functions of potential regulatory genes such as TFs. Our data suggest that hormonal interactions between auxin and ET may play a critical role in the regulation of onset of corolla senescence in petunia.

## Materials and methods

### Plant materials

All petunia plants were grown in a greenhouse at the University of California, Davis (USA), as described previously^3,21^.

*Petunia hybrida* ‘Mitchell Diploid’, a white flower cultivar, was used in the transcriptomic analysis. Corolla limbs were collected on day 0 (D0), when flowers were open but before the anthers dehisced, day 2 after anthesis (D2, corolla were fully expanded), day 4 (D4, corolla displayed a wilting sign at the tip edge, considered as the onset of flower senescence), and day 7 (D7, corolla showed wilting).

*P. hybrida* ‘Primetime Blue’, a purple flower hybrid cultivar, was used for VIGS analysis^22^.

### RNA preparation and Illumina sequencing

At least five corolla limbs were collected from five plants at random on each replicate. Corolla limbs from two independent biological replicates were used to extract total RNA and to sequence separately. The total RNA was obtained using the TRIzol method (Invitrogen, USA), combined with Ambion RiboPure™ Kit (Ambion, USA) to remove the contaminant DNA^3^. The purity and concentration of RNA were verified using NanoDrop™ 3100 Spectrophotometer (Thermo Scientific, USA) and Qubit RNA Assay Kit 2.0 (Life Technologies, CA, USA). The RNA integrity was examined by RNA Nano 6000 Assay Kit using the Agilent Bioanalyzer 2100 system (Agilent Technologies, CA, USA). Eight complementray DNA (cDNA) libraries were prepared using purified mRNAs. An Illumina HiSeq2000 machine was used to perform 100 paired-end sequencing according to the Illumina protocols. The raw data were deposited to NCBI as project number SRP124540.

### Sequence data processing and differential gene expression analysis

The quality of the raw reads was examined before and after trimming using FastQC software (http://www.bioinformatics.babraham.ac.uk/projects/fastqc/). Low-quality reads with a Phred quality score <20, sequences shorter than 40 bp, barcodes, polyA, polyT ends, and adapter sequences were removed. Clean reads from all samples were pooled and the read counts were normalized to the aligned FPKM (Fragments Per Kilobase of transcript per Million mapped reads) to quantify the gene expression level using cufflinks (version: 2.1.1)^23^. Differentially expressed genes (DEGs) were identified using Cuffdiff software (version: 2.1.1)^23^. The significance of DEGs was determined using fold change ≥2 or ≤0.5 as a cutoff^24^. False discovery rate (FDR) was adjusted across genes for significance levels (≤0.05) of all tests^25^.

Resulting sequences were mapped to the *P. axillaris* (one of *P. hybrida*’s parents) reference genome (https://solgenomics.net/organism/Petunia_axillaris/genome)^26^.

To obtain knowledge about expression profiles of DEGs throughout four development stages, the short time-series expression miner (STEM) was used to cluster DEGs^27^. The gene expression data and gene annotation files were uploaded to STEM. Expression profiles were analyzed using the STEM clustering algorithm^27^.

BiNGO 2.3 plugin tool in Cytoscape 3.2.1^28^ was used to gain knowledge about gene ontology (GO) terms of DEGs in each cluster. Over-represented GO terms were identified using a hypergeometric test with a significance threshold of 0.05 after Benjamini and Hochberg FDR correction^29^ with the whole annotated genome as the reference set^30,31^.

Mapman visualizationwas performed as described previously^32^ to identify gene families that may play essential roles in regulating corolla senescence. Contigs were classified into a set of hierarchical functional categories (BINs), using Mercator with a blast cutoff of 50^33^. Because one unigene might have multiple contigs, a functional term of a unigene was derived from its representative contig that had the highest bit score^34^. Enrichment analysis was completed through Fisher’s test using Mefisto (http://www.usadellab.org/cms/index.php?page=mefisto) with Bonferroni correction. Gene expression changes were viewed in Mapman 3.5.1R2^32^.

### VIGS plasmid construction and *Agrobacterium*-mediated infection

Tobacco rattle virus (TRV)-based VIGS^21^ was used to analyze the role of TFs in corolla senescence. TRV is a bipartite positive sense RNA virus with the TRV-RNA1 (pTRV1) and TRV-RNA2 (pTRV2)^35,36^. pTRV1 can replicate and move systemically without RNA2^35,36^. The pTRV2 was modified by introducing a chalcone synthase (*CHS*) fragment, which serves as a visual reporter for gene silencing to generate pTRV2/CHS vector for functional analysis of the genes-of-interest^21^. A gene specific fragment of each TF was amplified from petunia corolla cDNA using the corresponding specific primers listed in Supplementary Table S1, and then subcloned into the pTRV2/CHS vector to generate pTRV2/CHS-TF constructs. The constructs, pTRV1 and pTRV2/CHS (empty vector control) or pTRV2/CHS-TF were transformed into *Agrobacterium tumefaciens* strain GV3101 by electroporation^22^. The bacteria containing pTRV1 and pTRV2 (*TRV2/CHS* or *TRV2/CHS-TF-*) were mixed together in a 1:1 ratio immediately before inoculation^21,22^. The leaves of 4-week-old Primetime Blue seedlings were inoculated with the mixed bacterial culture using a 1 mL disposable syringe without a needle^21,22^. The inoculated seedlings were grown in a growth chamber under 16 h light/8 h dark (23/20 °C). The phenotypes of flowers were observed and monitored until silencing occurred, visualized as the purple anthocyanin pigment in the corollas turning white^21,22^. Flowers from wild-type, empty vector controls and TF-silenced petunias were used in longevity analysis.

### qRT-PCR analysis

One microgram of total RNA was reverse-transcribed using PrimeScript RT reagent with gDNA Eraser Kit (TaKaRa, Japan), according to the manufacturer’s instructions. Specific primers were designed by the Primer 3 program and listed in Supplementary Table S1. Amplifications were performed in an Applied Biosystems 7300 system (Applied Biosystems, USA). Melting curve analysis was performed and the absence of non-specific products and primer dimers were verified. For data analysis, average threshold cycle (*C*_T_) values were calculated for each gene of interest, on the basis of three independent biological samples and were normalized and used to calculate relative transcript levels as described elsewhere^37^. 26S ribosomal RNA was used as an internal standard for normalization^3^.

### ET measurement

ET emission was monitored using a laser-based ET detector (type ETD-300, Sense B·V., Nijmegen, The Netherlands) and a gas handling system (type VC-6, Sensor Sense B·V.) as described previously^3^. Briefly, flowers collected at D0 were placed into 70 ml sealed glass vials. The air was passed through a platinum-based catalyzer before entering the cuvettes in order to remove external ET and other hydrocarbons. A scrubber with KOH and CaCl_2_ was used to reduce the CO_2_ and the water content in the gas flow. ET emission was monitored and recorded in real time. Three biological replicates of every flowering stage were performed. Each experiment was repeated three times.

### Floral longevity

To measure longevity of intact flowers, white flowers from pTRV/CHS-TFs inoculated petunia ‘Primetime Blue’ plants were tagged at D0. The time when the corollas wilted and the edges collapsed was recorded^21^. At least 20 flowers of three plants from each of the three independent biological replicates were monitored. Purple flowers from water-inoculated wild-type and white flowers from pTRV/CHS-silenced (empty vector) plants were used as controls. Statistical analyses were performed using the SPSS package (Version 16.0; SPSS Inc., Chicago, IL, USA). One-way analysis of variance was performed for experiments with one independent variable. Duncan’s test was used as the *post hoc* test if significant differences were found.

## Results

### Floral senescence and ET production

**Fig. 1:**
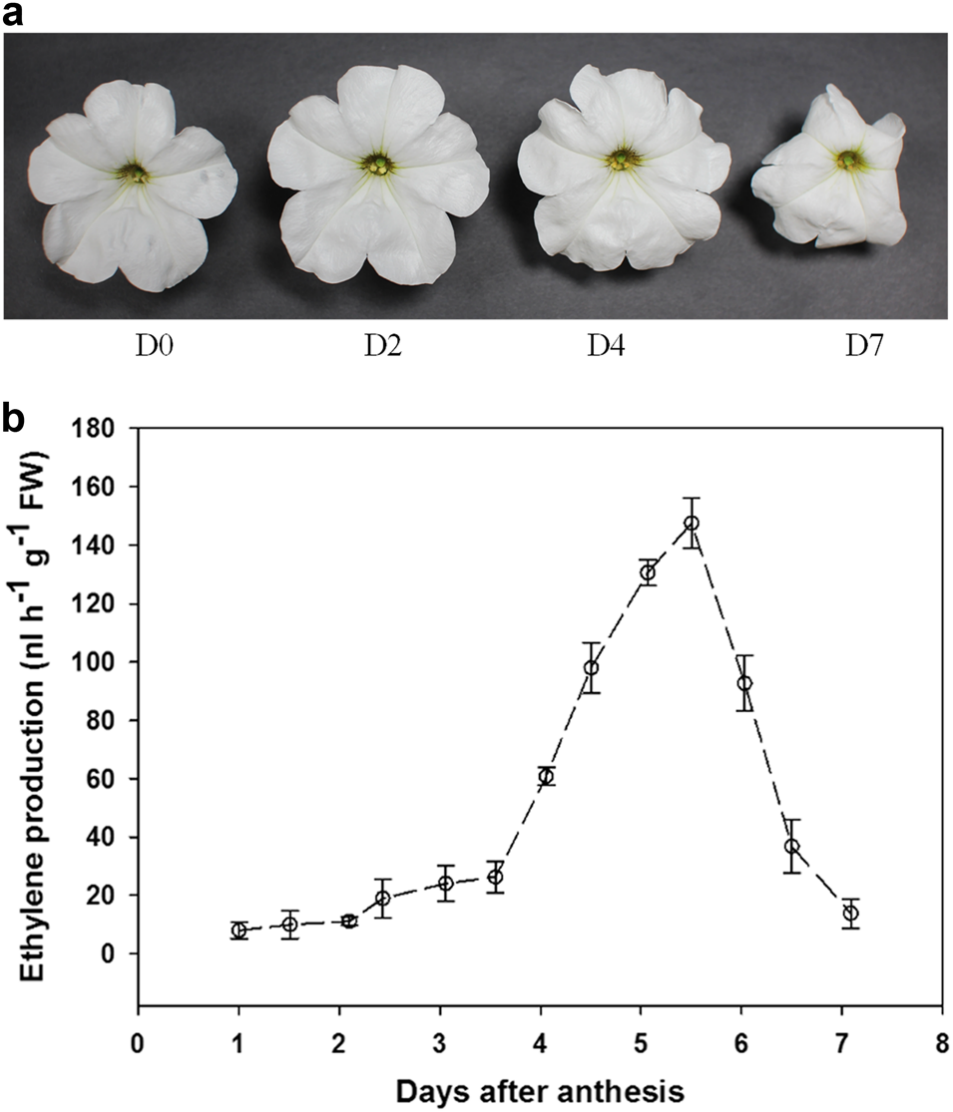
Natural senescence and ethylene production of wild-type (WT) *Petunia* × *hybrida* ‘Mitchell’ flowers. A representatives corolla phenotypes at different stages including corolla opening without anther dehiscence (D0), corolla expansion, 2 days after anthesis (D2), corolla with initial signs of senescence (D4), and wilting corolla (D7). B representatives ET production at various time points after corolla opening without anther dehiscence. ET emission was monitored using a laser-based ET detector (type ETD-300, Sense B.V., Nijmegen, The Netherlands) and a gas handling system (type VC-6, Sensor Sense B.V.). Three biological replicates of every flowering stage were performed. Values represent the average ± SD

Flowers that were fully opened but anthers not yet dehisced were marked as D0. The corollas continued to expand for 2 days. Visible senescence symptoms, such as curving of the corolla edges, were observed at an average of 4 days. Corolla wilting was found at about 7–8 days (Fig. 1a). We measured ET production using a real-time ET detection system, EDT-300. An increase and decrease of ET emission was detected during D2–D7 stage. The level spiked around D4, reaching the maximum level at 5.5 days, and then decreasing sharply (Fig. 1b).

### Dynamic transcriptome profiles during corolla development

**Fig. 2:**
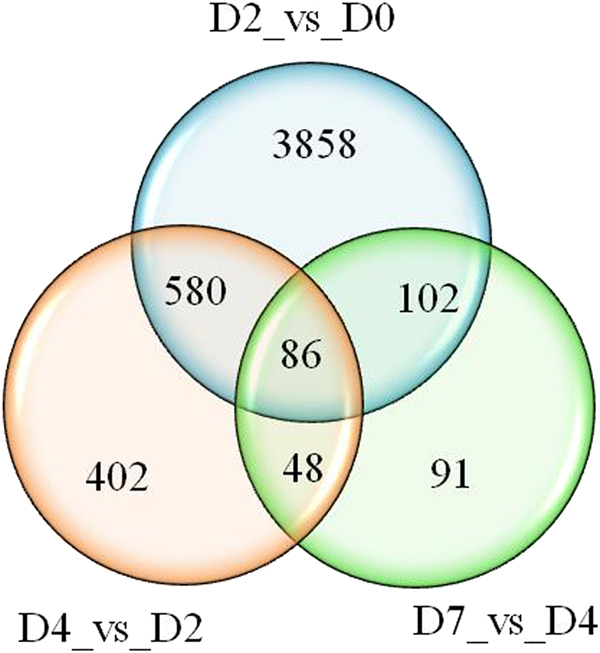
Unique and shared differential expression of unigenes in D0–D2, D2–D4, and D4–D7 pairwise analysis

In order to determine the alteration in gene expression during corolla senescence, we generated cDNA libraries composed of the samples collected from four developmental stages (D0, D2, D4, and D7) with two biological replicates. RNA sequencing of these libraries produced 49,421,030, 52,985,600, 47,813,446, and 56,552,704 clean reads at D0, D2, D4, and D7, respectively (Table 1). The sequences were mapped to the *P. axillaris* reference genome^26^ for annotation of all unigenes. The mapping rate was over 93% for samples of each stage (Table 1). Differential expression analysis was conducted by comparing four different developmental stages. Analysis on all four stages generated 5167 unigenes that were significantly differentially expressed across these stages. The number of DEGs was decreased from 4626 between D0 and D2, to 1116 between D2 and D4, and to 327 between D4 and D7 (Fig. 2).

**Table 1:** Statistics of annotation results for petunia unigenes

Sample	Clean reads	Raw reads	Mapping rate (%)
D0	49,421,030	52,430,622	93.67
D2	52,985,600	56,837,280	94.88
D4	47,813,446	50,671,186	93.26
D7	56,552,704	61,737,472	95.09

**Fig. 3:**
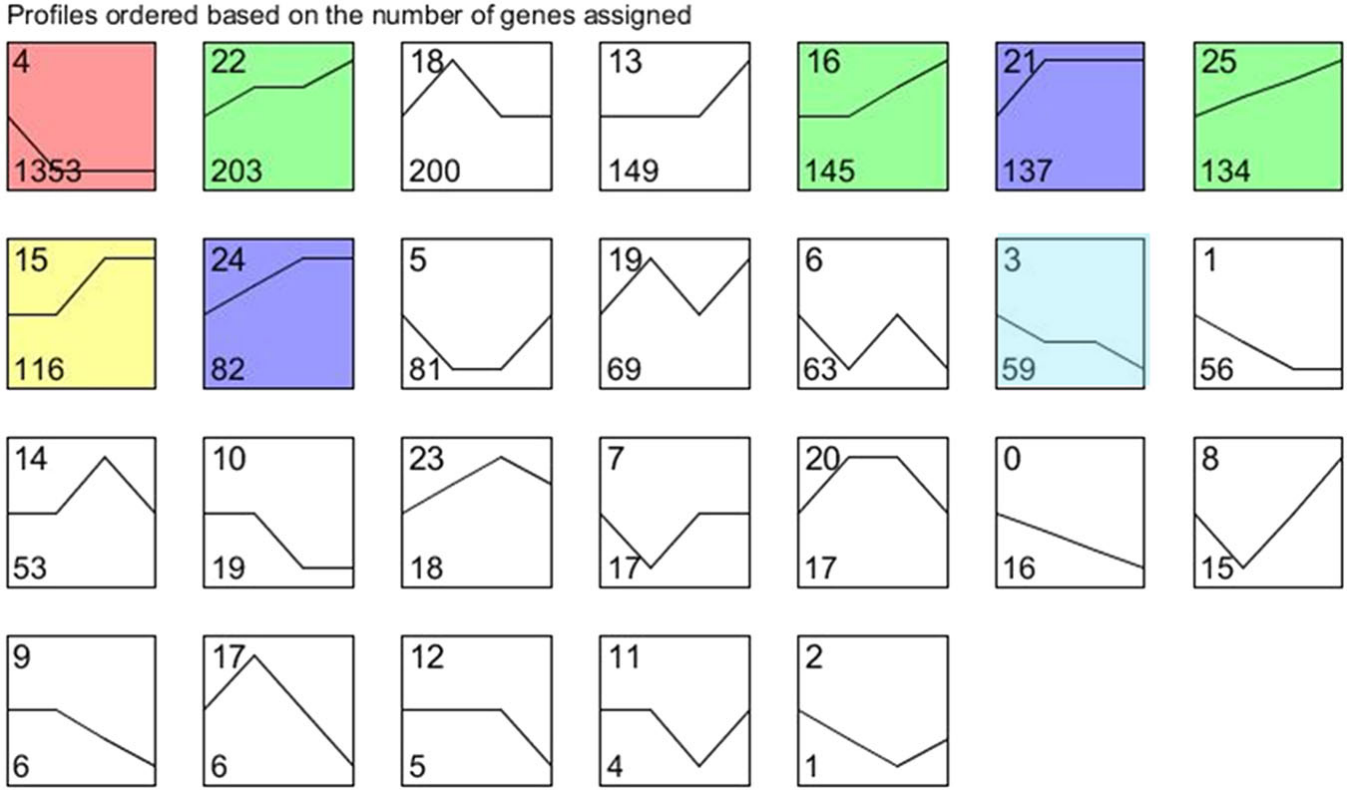
Cluster and STEM analysis of DEGs. Twenty-six clusters were obtained using STEM software. The colored clusters represented a significant level (*p* value ≤ 0.05). The number on the top is a cluster number. The number at the bottom is gene number assigned in each cluster

DEGs were clustered to generate expression patterns based on time series using the STEM software^27^. Cluster analysis of the data from four time points generated 26 clusters, including downregulated genes in clusters 0 through 12 and upregulated genes in clusters 13 through 25 (Fig. 3). A few clusters displayed a more complex pattern. For instance, clusters 2, 5, 7, 8, and 11 showed an initial decrease followed by upregulation. However, clusters 14 and 17 exhibited an initial increase followed by a decline (Fig. 3). In addition, the downregulated clusters 3 and 4 and the upregulated clusters 15, 16, 21, 24, and 25 were statistically significant (*P* ≤ 0.01) (Fig. 3).

### Identification of up and downregulated gene ontology at distinct time points

In order to identify up and downregulated GO at each selected time point, seven gene clusters exhibiting either significantly decreased (clusters 3 and 4) or increased (clusters 15, 16, 21, 24, 25) expression were further analyzed using Cytoscape software with its GO enrichment tool BiNGO^28^.

At the transition from D0 to D2, the metabolic processes of major macronutrients including ‘carbohydrates, lipids, aromatic amino acids, and nitrogen compounds’ were downregulated (cluster 4, Supplementary Figure S2). In addition, ‘cell wall organization and biogenesis’, ‘S-adenosylmethionine biosynthesis’, and ‘negative regulation of transcription, DNA-dependent’ and ‘RNA metabolism’ were downregulated (cluster 4, Supplementary Figure S2). However, ‘CK pathway’, ‘RNA modification’, ‘macromolecule methylation’, ‘DNA metabolism’, ‘ATP activity’, and ‘S-adenosylmethionine-dependent methyltransferase activity’ were upregulated (cluster 22, 24, and 25). At the transition from D2 to D4, ‘monosaccharide metabolism (hexose and glucose)’, ‘polysaccharide metabolism (glucan)’, ‘lipid catabolism’, ‘amino acid metabolism (including glycine, l-serine, and methionine)’, ‘S-adenosylmethionine biosynthesis’, and ‘l-phenylalanine biosynthesis’ were significantly upregulated (clusters 22 and 24, Supplementary Figure S2). ‘Response to auxin stimulus’ was also significantly upregulated (cluster 25). Downregulated GO terms were mainly ‘nicotianamine metabolism and biosynthesis’ (cluster 1, Supplementary Figure S2). Biological regulation (‘anion channel activity’ and ‘ion transmembrane transporter activity’) was over-represented among downregulated GO terms (cluster 3, Supplementary Figure S2). At the transition from D4 to D7, 'iron ion binding' was significantly upregulated. The only downregulated biological process was the auxin-mediated signaling pathway (cluster 3). The over-representation of 'ribosome and cytosolic small ribosomal subunit' was also enriched in the downregulated GO group (Supplementary Figure S2).

### Differential gene expression in hormone biosynthesis and signaling pathways

Hormone and transcriptional regulation pathways were found to be enriched in the significant cluster groups (cluster 16, 22, and 25). In order to further understand the key DEGs regulating corolla senescence, all DEGs related to hormone signaling and TFs across these three transitions were further analyzed with Mapman.

**Fig. 4:**
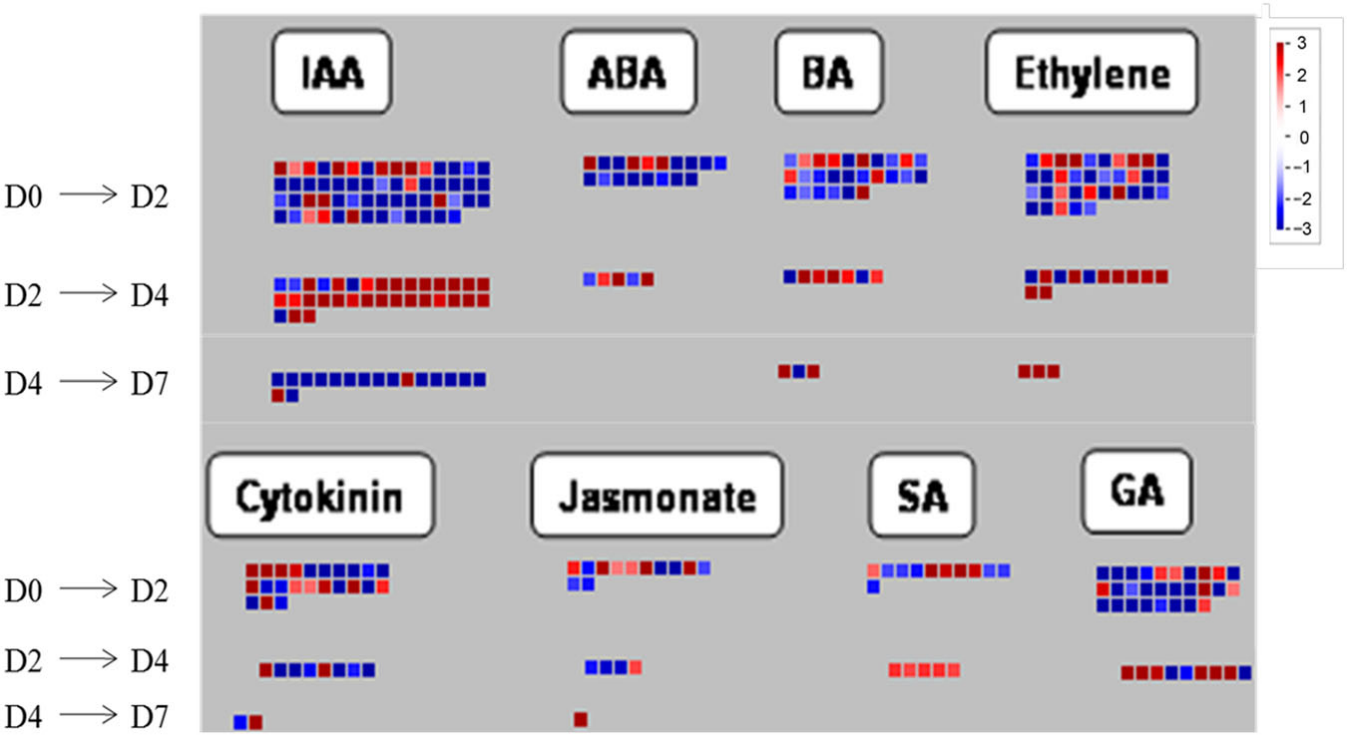
Display of gene expression involved in hormone biosynthesis and signaling pathway. Significantly DEGs (log_2_ fold changes (FC) ≥1, FDR ≤0.05) were visualized using Mapman software and organized into functional categories (BINs)^32,33^. Blue indicates a decrease and red an increase in gene expression (see color set scale on top right corner). Detailed information on each gene and its expression level is listed in Supplementary Table S2

Within the hormonal function term, we identified DEGs related to hormone biosynthesis and signaling pathways. Among these genes, the largest numbers of DEGs were involved in auxin and ET biosynthesis and signaling pathways, followed by DEGs in the GA pathway (Fig. 4). DEGs related to auxin were predominantly downregulated through the D0 to D2 (24 out of 27 DEGs) and D4 to D7 transitions (21 out of 27), but upregulated in the D2 and D4 transition (26 out of 27 DEGs) (Fig. 4). Among these DEGs, 17 *SAUR* and *SAUR-like* genes, three auxin-induced genes (Peaxi162scf01044g00226, Peaxi162scf00000g43013, and Peaxi162scf01133g00016), and two IAA-amido synthases were upregulated through the D2 to D4 transition (Supplementary Table S2). For the ET pathway, 37.5% of the DEGs (9/24) were upregulated through the D0 to D2 transition, and 45.8% of the DEGs (11/24) were upregulated through the D2 to D4 transition. DEGs relating to ET biosynthesis pathway such as *ACS* (Peaxi162scf00074g01725) and *ACO1* (Peaxi162scf00294g00812 and peaxi162scf00712g00513) were upregulated through the D2 to D4 transition, while *ACS10* (Peaxi162scf00620g00121) and *ACO* (Peaxi162scf00047g01927) were upregulated through the D0 to D2 as well as the D4 to D7 transitions (Supplementary Table S2).

DEGs related with GA pathway were upregulated in both the D0 to D2 and D2 to D4 transitions. Among these DEGs, *GA2OX8* (Peaxi162scf00082g02010 and Peaxi162scf00111g00920) and *GA3OX1* (Peaxi162scf00015g00525) were upregulated during petunia flower senescence (Fig. 4 and Supplementary Table S2). In addition, DEGs related to CK, ABA, JA, salicylic acid (SA), and 6-benzylaminopurine (6-BA) were obtained (Fig. 4 and Supplementary Table S2). Three genes related to the CK pathway were identified, including *CK oxidase 3*, *cytokinin-independent 1* (*CKI1*), and *CK response regulator 12* (*RR12*). These three genes were upregulated in the D0 to D2 transition (Fig. 4 and Supplementary Table S2). For the ABA biosynthesis pathway, *AAO3* (Peaxi162scf00217g00412) was upregulated at the D0 to D2 transition. *NCED4* (Peaxi162scf00045g00726) transcript was accumulated at the D2 to D4 transition. *ABA 8’-hydroxylase* (Peaxi162scf00045g00725) transcript was accumulated in the D2 to D4 and D4 to D7 transitions. Among those JA-related genes, *AOS* (Peaxi162scf00684g00578), *OPDA* (Peaxi162scf00688g00362), *LOX2* (Peaxi162scf00895g00019 and Peaxi162scf00895g00113), and *LOX3* (Peaxi162scf00038g02025) were upregulated at the D0 to D2 transition. No upregulated DEGs related to JA biosynthesis were detected at the D2 to D4 transition (Supplementary Table S2). For the SA pathway, two UDP-glucosyltransferase genes, *UGT74E2* (Peaxi162scf00883g00811) and *UGT74F1* (Peaxi162scf00303g00048), were upregulated at the D0 to D2 transition. SABATH methyltransferase was upregulated at the D2 to D4 transition. No DEGs involved in the SA pathway were detected at the D4 to D7 transition (Fig. 4 and Table Supplementary S2). In the 6-BA pathway, identified DEGs were mostly members of cytochrome P450 family (Supplementary Table S2).

### Differential expression related to transcription factors

**Fig. 5:**
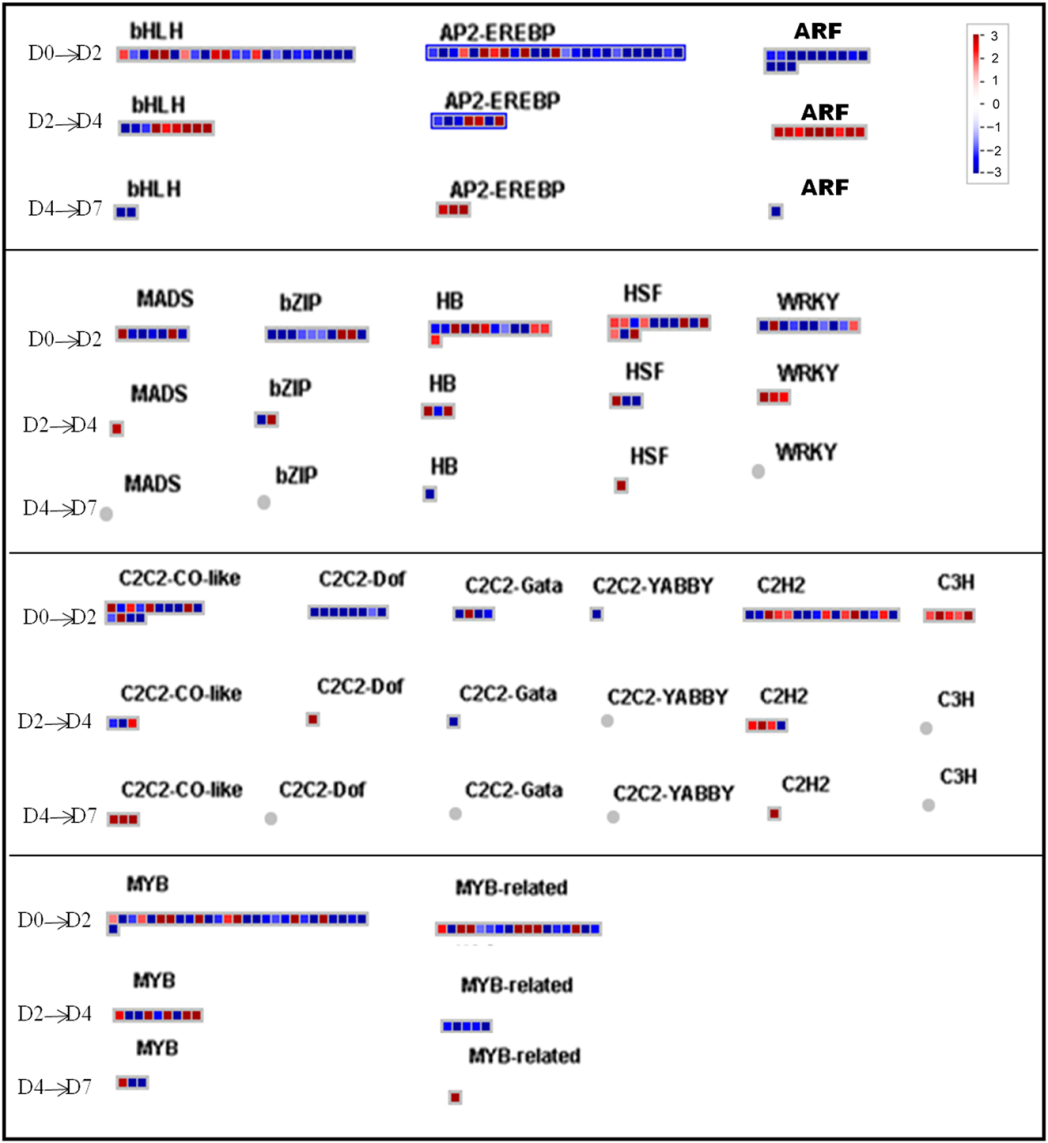
Display of gene expression of TFs. Significantly DEGs (log_2_ fold changes (FC) ≥1, FDR ≤0.05) were visualized using Mapman software and organized into functional categories (BINs)^32,33^. Blue indicates a decrease and red an increase gene expression (see color set scale on top right corner). Detailed information on each gene and its expression level is listed in Supplementary Table S3

The differential expression of specific TFs at specific time points during corolla senescence was analyzed with Mapman (Fig. 5). A total of 409 DEGs encoding TFs was observed at D2, with 175 upregulated DEGs. Major TF families included AP2-EREBP, ARF, bHLH, HSF, MADS-box, MYB, WRKY and C2C2-CO-like (Fig. 5 and Table S3). Among all the differentially expressed TFs, ERF was the predominant family with 25 DEGs, including 14 upregulated genes at the D2 to D4 transition, followed by the zing-finger (15/24), ARF (9/15), MYB (9/26), bHLH (8/18), and HD-ZIP (5/11) families (Fig. 5 and Supplementary Table S3).

### Verification of RNA-seq results with qRT-PCR

**Fig. 6:**
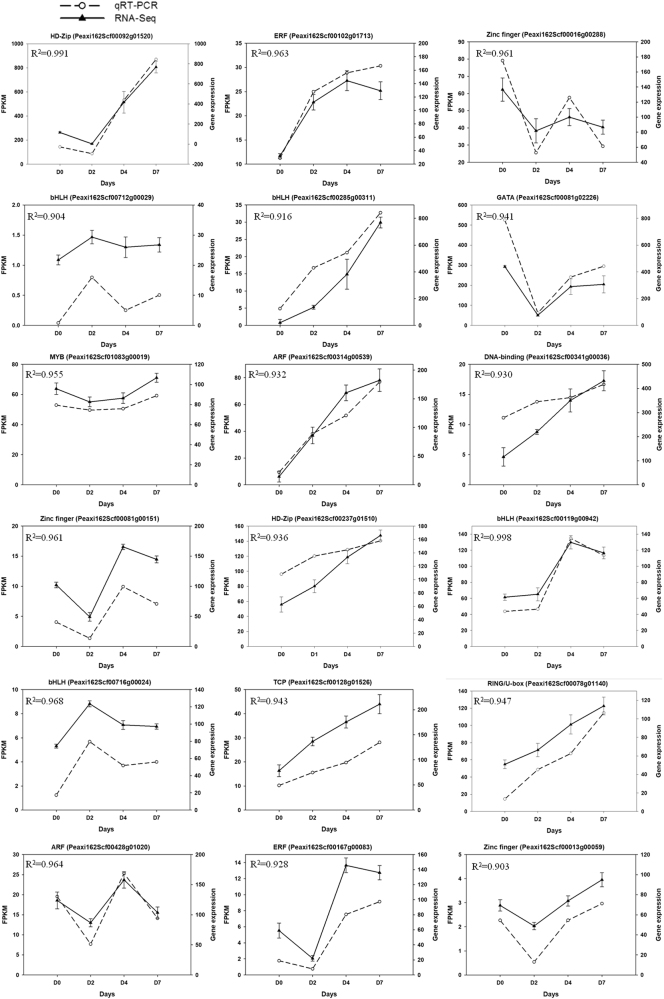
Validation of RNA-seq data by qRT-PCR. Eighteen genes were selected and their time-course expression profiles were evaluated at specific time points. cDNA analysis was performed by quantitative reverse transcription-polymerase chain reaction (qRT-PCR) amplification with specific primers designed by PRIMER 3 (Table S1). Transcript levels were normalized to 26S rRNA. Data represent three independent replicates (SD, *n* = 5). The correlation coefficient between RNA-seq and qRT-PCR was listed on the left corner of each gene expression figure

In order to verify our RNA-seq data, 18 differentially expressed TFs were randomly selected for qRT-PCR (Fig. 6). The correlation coefficients between RNA-seq and qRT-PCR were calculated. Overall, the qRT-PCR data were in agreement with the DEGs results. The pairwise correlation coefficient is higher than 0.90. The results showed that RNA-seq for counting transcripts reflects transcript abundance and can be used for gene expression analysis.

### Silencing TFs affects floral longevity

Although many TFs show differential expression during corolla senescence, few experiments employing silencing or over-expression have been conducted to confirm the regulatory function of TFs. We used TRV-based VIGS technology to examine the role of TFs in corolla senescence (Table 2). The results showed that the inhibition of some TFs prolonged the longevity of corolla in silenced flowers compared with wild-type (WT) and empty vector controls. Among these selected TFs, plants with the silenced gene encoding MADS-box (Peaxi162Scf01084g00119) significantly extended floral longevity by >3 days on unpollinated intact flowers (Table 2, experiment 4). Silencing *ERF* (Peaxi162Scf00167g00083) and *bHLH* (Peaxi162Scf00285g00311) extended longevity by >2 days. These increases in longevity were substantial compared with WT flowers and empty vector controls. Moreover, suppressed expression of Zinc finger (Peaxi162Scf00081g00151, Peaxi162Scf00105g01114, Peaxi162Scf00016g00288, and Peaxi162Scf00013g00059), *ERF* (Peaxi162Scf00024g00271), *HD-Zip* (Peaxi162Scf00092g01520), *bHLH* (Peaxi162Scf00285g00311), and *ACBF* (Peaxi162Scf01105g00218) TFs extended floral longevity by >1 day (Table 2, experiment 1). On the other hand, silencing of some TFs shortened the corolla longevity compared with WT and empty vector controls, including *ARF* (Peaxi162Scf00314g00539) (Table 2, experiment 1), *bZIP* (Peaxi162Scf00285g00011) (Table 2, experiment 3), and several members of the zinc finger family (Peaxi162scf00013g00084 and Peaxi162Scf00060g00211) (Table 2, experiment 1).

**Table 2:** Longevity of unpollinated TFs silenced flowers on petunia

Gene ID	Flower longevity (days ± SD)	Description	Increased/decreased days compared to WT	Increased/decreased rate of longevity compared to WT (%)	Reference
*Experiment 1*					
Peaxi162Scf00081g00151	9.89 ± 1.43c	Zinc finger	1.47*	17.46	
Peaxi162scf00013g00084	7.04 ± 1.08b	Zinc finger	−1.38	−16.39	
Peaxi162Scf00060g00211	7.93 ± 1.48b	Zinc finger	−0.49	−5.82	
Peaxi162Scf00105g01114	9.5 ± 1.57c	Zinc finger	1.08*	12.83	
Peaxi162Scf00207g01365	9.17 ± 0.75c	Zinc finger	0.75	8.91	
Peaxi162Scf00003g00267	8.5 ± 1.06a	Zinc finger	0.08	0.95	
Peaxi162Scf00016g00288	9.95 ± 1.29c	Zinc finger	1.53*	18.17	
Peaxi162Scf00013g00059	9.63 ± 0.69c	Zinc finger	1.21*	14.37	
Peaxi162Scf00167g00083	10.7 ± 1.19d	ERF	2.28**	27.08	
Peaxi162Scf00024g00271	9.64 ± 0.55b	ERF	1.22*	14.49	
Peaxi162Scf00102g01713	8 ± 0.87a	ERF	−0.42	−4.99	
Peaxi162Scf00092g01520	9.67 ± 1.68c	Homeobox	1.25*	14.85	
Peaxi162Scf00237g01510	10.15 ± 1.55d	Homeobox	1.73*	20.55	[22]
Peaxi162Scf00285g00311	11.36 ± 1.23d	bHLH	2.94**	34.92	[51]
Peaxi162Scf00712g00029	9.42 ± 1.46c	bHLH	1*	11.88	
Peaxi162Scf00119g00942	8.41 ± 1.22a	bHLH	−0.01	−0.12	
Peaxi162Scf00428g01020	8.35 ± 0.77a	bHLH	−0.07	−0.83	
Peaxi162Scf00081g02226	8.79 ± 1.07a	LIM	0.37	4.39	
Peaxi162Scf01083g00019	10.25 ± 1.6d	MYB	1.83*	21.73	
Peaxi162Scf00420g00728	8.4 ± 1.17a	NAM	−0.02	−0.24	
Peaxi162Scf00045g01824	8.73 ± 1.49a	CO-like	0.31	3.68	
Peaxi162Scf00314g00539	7.27 ± 0.88b	ARF2	−1.15	−13.66	
Peaxi162Scf00341g00036	9.7 ± 1.46c	DNA binding	1.28*	15.20	
TRV CHS white flowers	8.68 ± 1.19a	Control 1			
WT purple flowers	8.42 ± 1.14a	Control 2			
*Experiment 2*					
Peaxi162Scf01105g00218	9.6 ± 0.77a	NAM	0.1	1.05	
Peaxi162Scf00051g01410	10 ± 1.15b	NAM	0.5	5.26	
TRV CHS white flowers	9.35 ± 0.75a	Control 1			
WT purple flowers	9.5 ± 0.79a	Control 2			
*Experiment 3*					
Peaxi162Scf00998g00127	7.96 ± 0.96a	NAM	0.75	10.40	[3]
Peaxi162Scf00287g00193	8.5 ± 1.89a	ARF19	1.29*	17.89	
Peaxi162Scf00285g00011	5.6 ± 0.68b	bZIP	−1.61	−22.33	[54]
TRV CHS white flowers	7.79 ± 0.92a	Control 1			
WT purple flowers	7.21 ± 0.95a	Control 2			
*Experiment 4*					
Peaxi162Scf00002g00037	9.8 ± 1.13^a^	MYB	0.75	8.29	
Peaxi162Scf00716g00024	9.07 ± 1.28^a^	bHLH	0.02	0.22	
Peaxi162Scf00128g01526	9.88 ± 1.38a	TCP	0.83	9.17	
Peaxi162Scf01084g00119	12.8 ± 2.9^b^	MADS	3.75***	41.44	
TRV CHS white flowers	9.15 ± 1.55a	Control 1			
WT purple flowers	9.05 ± 1.23a	Control 2			

Petunia plants were infected with *Agrobacterium* transformed with pTRV2/CHS as an empty vector control (named TRV CHS white flowers) or pTRV2/CHS-TF vector (displayed as Gene ID). WT purple flowers as an uninfected control. For each line, 10 corollas from each of three individual plants were used for testing flower longevity. The recorded longevity represents the time from flower full open but before anthers dehiscing to complete wilting of the corolla. Flower longevity data (means ± SD, *n* = 10) followed by different letters are significantly different at *P = *0.05 within a given experiment

Notes: *: extending 1 day more than WT; **: extending 2 days more than WT; ***: extending 3 days more than WT; −: days and % of WT decreased

## Discussion

### Onset and execution of corolla senescence in ET-sensitive petunia

ET is a key flower senescence promoting hormone in ET-sensitive species^1,3^. In this study, transcriptional dynamics at four distinct developmental stages of corolla in petunia were monitored. The ‘S-adenosylmethionine biosynthesis’ GO term was significantly upregulated at the D2 and D4 transition (Supplementary Figure S2). Expression of *ACS* and *ACO* genes was upregulated through D2 to D4 and D4 to D7 transitions. The increase of ET emission was initially detected at the D2 to D4 transition, while the spike of ET emission occurred at the D4 to D7 transition (Fig. 1b). These data suggest that early onset of corolla senescence may occur in the transition from D2 to D4, and execution of senescence takes place in the transition from D4 to D7.

### Roles of the auxin signaling pathway during corolla senescence

Notably, large alterations in abundances of auxin-related transcripts occurred throughout the four developmental stages, especially through the transition from D2 to D4. Although, at present, the role of auxin in plant senescence remain poorly defined, and contrasting observations have been obtained from different species. Several studies have reported an involvement of auxin in the process of senescence, especially in petal senescence. For example, in cut carnation flowers, exogenous application of IAA hastened the rise in ET production and flower wilting^14^. 2,4-dichlorophenoxyacetic acid (2,4-d), a synthetic auxin, induced the expression of ACC synthase genes in the styles, ovaries, and petals^15^. It was reported that in the corollas of pollinated petunias, ‘response to auxin stimulus’ and ‘response to ACC’ were significantly enriched at 12 hap^16^. Interaction between auxin and ET occurred at the early stage of pollination^16^. Furthermore, the interaction between ET and auxin was also reported in ET-induced corolla senescence in petunia^20^. Interestingly, during pear ripening, the auxin-associated transcripts are significantly upregulated in the S2 to S3 transition (106–113 days after full bloom, when fruit gained the ability to soften after ET treatment) before pear ripening and downregulated in the S3 to S4 transition (113–120 days after full bloom, when fruit developed the capacity to soften without ET treatment)^34^. In addition, auxin level declined prior to ripening in tomato, grape, and strawberry fruit^38,39^. Moreover, the largest number of DEGs related to auxin were observed in the abscission process of rose petal^40^. Downregulation of *RhIAA16* by VIGS in rose promoted petal abscission^40^. In our transcriptome data, DEGs in the auxin pathway, including auxin-responsive genes (SAUR-like genes), auxin-induced genes (IAA13), and auxin efflux carrier were all induced at the D2 to D4 transition, where ET production was increased. However, those auxin-related genes were downregulated in the D4 to D7 transition (Supplementary Table S2), while expression of *ACO* and *ACS* genes was upregulated (Supplementary Table S2) and ET production reached a peak at 5.5 days (Fig. 1b). Taken together, we postulate that auxin might play common and vital positive roles in activating ET production and regulating developmental process that lead to subsequent attainment of ripening, senescence, and abscission capacity.

### Other hormone changes in petunia corolla senescence

Changes in ABA levels during petal senescence depend on the species. For example, during petal senescence in cut carnations, there is a small increase in ABA level^41^. In *P.* × *hybrida* cv. Ultra (Blue) and cv. Primetime (purple), the ABA level was decreased at the initial senescence stage and then increased at the late stage. However, in *P.* × *hybrida* cv. Dreams Appleblossom (pink), the ABA level increased as senescence progressed^9^. Proteomic analysis for ET-treated petunia corolla suggested that the external ET increased the protein level of PP2C and SnRK2A^20^. In our experiments, the upregulated expression of genes related to ABA biosynthesis suggests that the ABA biosynthesis pathway is involved in ET-dependent petal senescence.

In ET-dependent floral senescence, the role of CK is still unclear. The endogenous CK level in petal senescence varies with the species. CK levels are decreased during corolla development and senescence in carnation, roses and cosmos^1^. However, CK levels showed different patterns in petunia, such as an increase during floral development and a decrease at the late stages of senescence^42,43^. Exogenous application of 6-BA, prolonged flower life and repressed ET biosynthesis and perception gene expressions^43^. In *Hibiscus rosa-sinensis*, the CK-related transcripts had a higher abundance in an earlier stage than a later stage of flower senescence^44^. Our results also showed that three identified DEGs related to CK biosynthesis were induced at the earlier stage of corolla development. Therefore, the CK signaling pathway might play a role in an earlier development stage of corolla. In the SA pathway, identified DEGs included one UGT74E2 (Peaxi162scf00883g00811), two UGT74F1 (Peaxi162scf00303g00048 and Peaxi162scf00045g01732), and a TPR-like gene (Peaxi162scf00265g00033). UGT74E2 are strongly induced by H_2_O_2_ and may allow integration of ROS and auxin signaling^45^.

### Transcription factors regulate corolla senescence in petunia

Expression changes of TFs have profound effects on flower longevity. Many members of ERFs, ARF, NAC, bZIP, HD-Zip, and bHLH families were identified during pollination-induced^16^ and ET-induced corolla senescence^3^. However, there is little research on the roles of TFs in flower longevity. Our VIGS experiments provided valuable data for further investigation of the functional role and interactions of these TFs for regulating flower senescence. ERF2 in the ET signaling pathway is highly upregulated in senescing daffodil corollas^18^. In our experiments, two upregulated ERFs (Peaxi162Scf00024g00271 and Peaxi162Scf00050g00086) were silenced using TRV-based VIGS system, resulting in significantly extended flower longevity (Table 2). Notably, expression of most auxin-related genes, such as ARF genes, was upregulated at the D2 to D4 transition. Interestingly, *Small Auxin Up RNA 36* (*SAUR36*) is upregulated during leaf senescence and acts as a positive regulator of leaf senescence in *Arabidopsis*^46^. *Auxin Response Factor 2* (*ARF2*) positively regulated leaf senescence. Transcript abundance of *ARF2*, together with *ARF7/NPH4* and *ARF19*, is highly accumulated in senescing *Arabidopsis* leaves^47^. In *Arabidopsis*, *arf2* mutant displays delayed leaf senescence^47^. Our VIGS experiments showed that silencing *ARF19* (Peaxi162Scf00287g00193) prolonged flower longevity, but silencing *ARF2* (Peaxi162Scf00314g00539) caused premature corolla senescence. Taken all together, we postulate that genes related to auxin play either positive or negative roles in regulating corolla senescence. A recent study has illustrated that *AINTEGUMENTA* (*ANT*), a member of the AP2/ERF TF family functioning downstream of *ARF2* in *Arabidopsis*, negatively regulated leaf senescence^48^. The underlying mechanisms how ARFs regulate onset of corolla senescence require further studies in the future. With the exception of ARF, the MADS-box gene (Peaxi162Scf01084g00119) was upregulated in the corolla senescence process in petunia. Silencing this gene substantially extended the flower longevity (Table 2). It would be very interesting to investigate how the MADS-box protein regulates flower senescence although it is beyond the scope in this study.

In addition, significant upregulation of TFs, including bHLH, HD-Zip, and C2C2-CO-like, was observed. Recent reports have demonstrated that bHLHs are involved in petunia petal senescence. For example, a bHLH TF *ANTHOCYANIN1 (AN1*) has been showed to regulate petal senescence in petunia^49^. In our experiments, four bHLHs were silenced by VIGS (Peaxi162Scf00119g00942, Peaxi162Scf00285g00311, and Peaxi162Scf00712g00029), resulting in extended flower longevity (Table 2, experiment 1). Interestingly, over-expression of a bHLH (Peaxi162Scf00285g00311, *PhFBH4*) hastened petal senescence by modulating the expression of the ET biosynthesis gene *ACO*^50^. HD-Zip TFs have been reported to be involved in ABA- and ET-induced senescence in rose petals^51^. Chang et al.^22^ demonstrated that silencing *PhHD-Zip* reduced the expression of ET biosynthesis-related genes (*ACO1*, *ACO4*, and *AC*S) and ET production. In our study, petunia plants with VIGS-silenced HD-Zip TFs (Peaxi162Scf00092g01520 and Peaxi162Scf00237g01510) extended flower longevity (Table 2, experiment 1 and 2). Furthermore, ET treatment induced the transcript abundance of *PhHD-Zip* [22]. These results suggest that HD-Zip TFs might be involved in petal senescence through regulating ET biosynthesis. NAC TFs were upregulated during senescence of petunia petal. Our previous study demonstrated that multiple NAC TFs were downregulated in ET-insensitive petunia petals^3^. In *Arabidopsis*, a NAC TF, *AtNAC092*, is induced through *ETHYLENE INSENSITIVE 2* (*EIN2*) and *EIN3*^52^ during leaf senescence. However, silencing three selected NAC TFs (Peaxi162Scf00051g01410, Peaxi162Scf01105g00218, and Peaxi162Scf00998g00127), lightly increased flower longevity (Table 2, Experiment 2). Interaction of senescence-associated NAC TFs expression and ET signals needs to be further clarified in ET-sensitive petal senescence.

Notably, some members of other putative TFs that share high similarity with MYB-DNA-binding proteins, bZIPs, and zinc fingers were downregulated. The petunia with VIGS-silenced zinc finger TF (Peaxi162scf00013g00084) had shortened flower longevity (Table 2, experiment 1). SUF4, encoding a zinc finger superfamily protein, was downregulated in the transgenic ET-insensitivity *etr1-1* petunia^3^. The differential expression of bZIP was identified in pollinated petal senescence^16^. Flowers with overexpressed bZIP (Peaxi162Scf00285g00011) have been shown to have delayed senescence while flowers with silenced bZIP have shortened longevity^53^. The relationship between these TFs and ET needs to be clarified in the future.

We would like to point out that there were many downregulated DEGs that may play critical roles in the regulation of flower senescence. Study on the downregulated DEGs using upregulation approaches or CRISPR/Cas9 technology will be valuable and informative in the future.

At present, most research has applied heterologous genes to modify traits of ornamental plants^54^. Modifying the ET pathway both directly and indirectly extended flower longevity^55^. In summary, hormone pathways, especially the crosstalk between auxin and ET, and transcriptional regulation play a vital role in ET-sensitive corolla senescence. Silencing or overexpressing several genes associated with senescence simultaneously is needed to further manipulate flower longevity in the future. Combined with the RNA-seq data, functional analysis using a relative high-throughput VIGS system provides valuable data and generates a list of promising targets for further investigation and future plant breeding in the flower industry.

## Electronic supplementary material

Supplemental Figure S1 2

Supplemental Table S1, S2 and S3

Supplemental Table S4
