# Supplementary material for: Transcriptome profiling reveals regulatory mechanisms underlying corolla senescence in petunia
Source: Hortic Res. 2018 Apr 1;5:16. doi: 10.1038/s41438-018-0018-1 (PMC5878830; doi:10.1038/s41438-018-0018-1)

Supplemental Figure S1. Flow chart for RNA-seq analysis

**Short Time-series Expression Miner**

**Mapman Analysis**

**Expression in transcript level**

**Alignment with reference**

**Clean reads (fastq)**

**Differential expression**

**Raw reads (fastq)**

**GO Enrichment**

**KEGG Enrichment**

Supplemental Figure S2 Metabolic processes and cellular component activated or repressed at different time points during corolla senescence.

The overrepresented GO terms for the combined clusters of genes either up-regulated (clusters 15,16, 21, 22, 24 and 25, 817 genes) or down-regulated (clusters 4 and 3, 1412 genes) during corolla senescence were enriched using BiNGO^28,30^. Significant up-regulated or down-regulated GO terms at each time point during corolla senescence are indicated.


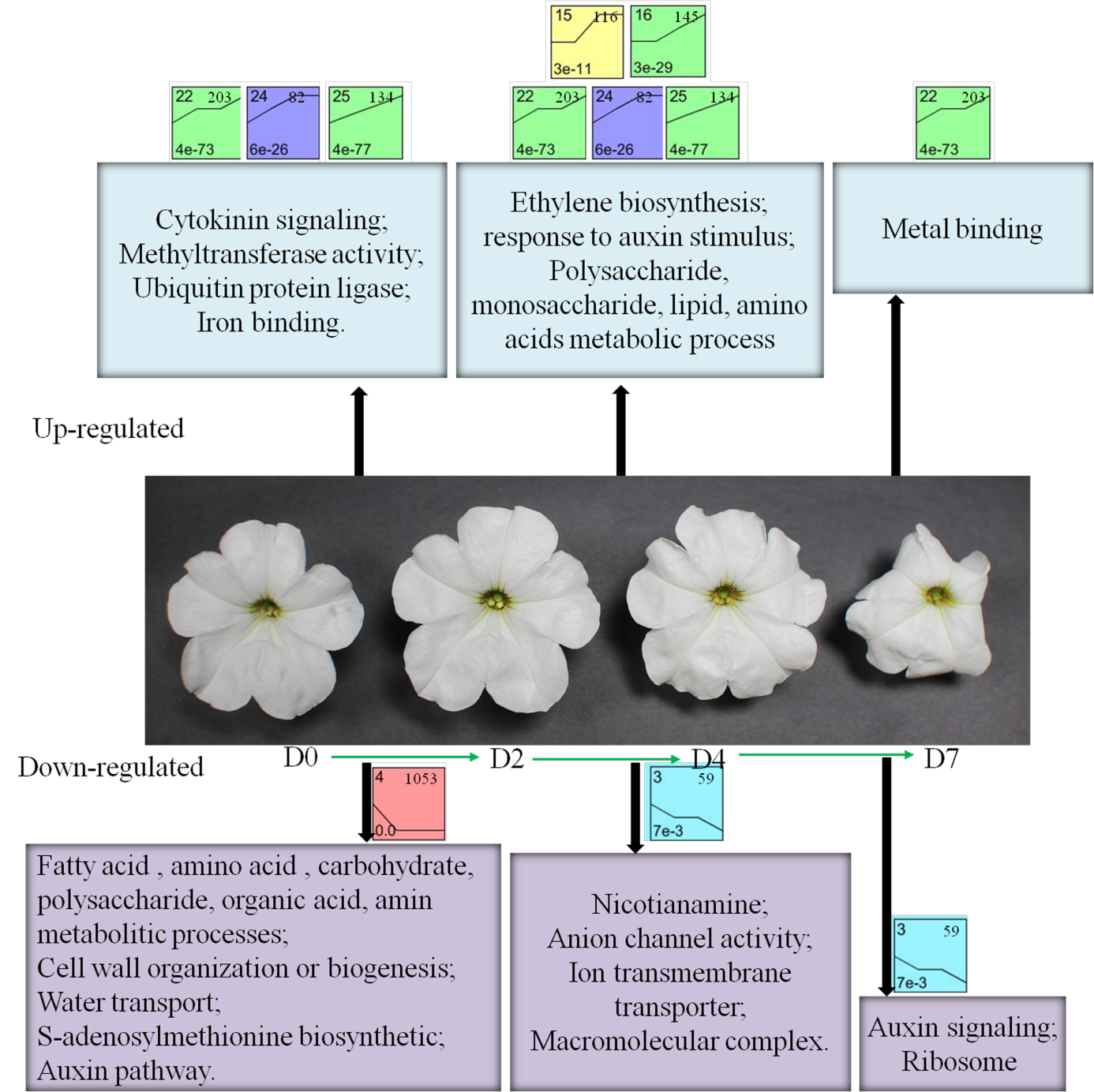

Supplement: Supplementary file 1 — Supplemental Figure S1 2 [file 41438_2018_18_MOESM1_ESM.docx]
