# Supplementary material for: Transcriptome profiling reveals regulatory mechanisms underlying corolla senescence in petunia
Source: Hortic Res. 2018 Apr 1;5:16. doi: 10.1038/s41438-018-0018-1 (PMC5878830; doi:10.1038/s41438-018-0018-1)
Supplement: Supplementary file 2 — Supplemental Table S1, S2 and S3 [file 41438_2018_18_MOESM2_ESM.docx]

**Supplementary** Table S1. Primer sequences used for amplifying fragments of each transcription factors

| Gene ID | Gene specific primers (5’- 3’) |
| --- | --- |
| Peaxi162scf00013g00084 | Forward: TGTGGTCATGTCTAATGTTAGAAGC  Reverse: CAATTGGTAGCATCTCTCTCACC |
| Peaxi162Scf00060g00211 | Forward: GCATAACAACCATGGCAAATTAC  Reverse: TCATATGTCCTCCTAAAGCTTGC |
| Peaxi162Scf00167g00083 | Forward: CACATTCACCAGTTTCCCTTTAC  Reverse: AGCTCTAGCAGCATCCATATCAG |
| Peaxi162Scf00081g00151 | Forward: TTTGTACAAGTGTTCCGTTTGTG  Reverse: CCAACACTTACACTTCCGTTACC |
| Peaxi162Scf00105g01114 | Forward: TGGGAAGATATTTCTGTGAAACC  Reverse: ATCTCCTTCAAGCAGTTCTTGTG |
| Peaxi162Scf00092g01520 | Forward: GTTGGAGACATTAAACGAACAGC  Reverse: CCAGTGTTCAGGTGATTCTAAGG |
| Peaxi162Scf00207g01365 | Forward: TACCATGGAAACTCAAGAAGAGG  Reverse: TAGCTGATCTTGCACGTGTCTAC |
| Peaxi162Scf00003g00267 | Forward: CAACTCCCACTACTATAACCAACG  Reverse: TCATAGTGACGACGTTTATGTCC |
| Peaxi162Scf00024g00271 | Forward: AGTTCTTGAATTTGAATGCTTGG  Reverse: TCAGCATGTTCATCACCAAATAG |
| Peaxi162Scf00102g01713 | Forward: GATGTGATTTCACCAAGCATTTC  Reverse: GTAGGTCAAAGGTTCAAGTCACG |
| Peaxi162Scf00016g00288 | Forward: TTCTTATCAAGCTCTTGGTGGAC  Reverse: CACCTAGTTTGCCTTCATAGTGG |
| Peaxi162Scf00285g00311 | Forward: CTCGAATCAGTGAAAGAATGAGG  Reverse: CTTGTTGTTTGAGACCTTCTTGG |
| Peaxi162Scf00081g02226 | Forward: TCCTTTGAGGGAGTTCTTTACTG  Reverse: GTAGTTGGAAGGGCTGATTACAC |
| Peaxi162Scf01083g00019 | Forward: GGTTCGATTAACAGATGGGTTG  Reverse: CGCCAATCACCTTTACCAAG |
| Peaxi162Scf00314g00539 | Forward: ATAGCATGCATCTTGGGTGTACT  Reverse: TCAGACATCTCCATTTAGATTCCA |
| Peaxi162Scf00712g00029 | Forward: TAAATCTAGTGGCCTCCTGAAAC  Reverse: GGCCTCAACTCTACTACACCATTAC |
| Peaxi162Scf00420g00728 | Forward: TTTGCCAGCCTTAATCATCC  Reverse: TACTGGTGCATAACCCAACG |
| Peaxi162Scf01105g00218 | Forward: GGCAGATGAAGAATTAGTACGG  Reverse: GCCCTATCCTTTCCAGTTGTC |
| Peaxi162Scf00237g01510 | Forward: TGTGAAGATGGAAGAGCACAA  Reverse: CCAGACATTTTCTTTATTCGG |
| Peaxi162Scf00119g00942 | Forward: TTGACCTGGAACCTGAAAGTC  Reverse: CCAGACTACTCGCTGAAGTGC |
| Peaxi162Scf00341g00036 | Forward: TCGTTGGAGGTAGTCAACAGG  Reverse: GTGTCCATGTTAGGTCACTGC |
| Peaxi162Scf00045g01824 | Forward: GTCTCTTCATCATCTATGGAAGTGG  Reverse: TCGATACCTCAACACCCTTG |
| Peaxi162Scf00013g00059 | Forward: GGAGTAGCCACAAACATGAGC  Reverse: CATGGTAGCAGACACGAATCC |
| Peaxi162Scf00428g01020 | Forward: GAGATGCCAATAGTTCTGGTG  Reverse: TCCCAACTTCTCCCATTACATC |
| Peaxi162Scf00051g01410 | Forward: AAGGCATTGGTGTTTTACGC  Reverse: GAGGTGGTAGCGGTGGTAGA |
| Peaxi162Scf00002g00037 | Forward: AGCTGCCTACTGGAAGCTGA  Reverse: ATCCGTGCCTGTTCTCTACG |
| Peaxi162Scf00716g00024 | Forward: CAGCGCACACTGTTGAATCT  Reverse: TAGAACCGCTGGTTCAGCTT |
| Peaxi162Scf00128g01526 | Forward: TTGACTCGTGAATTGGGTCA  Reverse: GGCATCTGGGGTCTACTTGA |
| Peaxi162Scf01084g00119 | Forward: TTGTGATGCCAAAGTTTCCA  Reverse: GCTTCCTCAGTTGCTCTTGC |
| Peaxi162Scf00998g00127 | Forward: TTTCTCTTCCACCACTCCTTGAG  Reverse: CCACCTGAGAATAACGGAAGATG |
| Peaxi162Scf00287g00193 | Forward: TCAGCTAGCCTAGCAGATTCTC  Reverse: GATCCTTGGGAGGAATTTGTTGG |
| Peaxi162Scf00285g00011 | Forward: AACATGACTACAACACAATATGC  Reverse: GGCTTCAGCTGATGTTCTATC |

**Supplementary** Table S2 Unigenes associated with hormone biosynthesis and signaling pathway exhibiting a Log2 FC≥1 and p≤0.05 in at least one transition.

| **Gene ID** | **FCD2/D0** | **FCD4/D2** | **FCD7/D4** | **Description** |
| --- | --- | --- | --- | --- |
| **ETH** | | | | |
| **peaxi162scf00024g01820** | -3.74 | 5.21 | 0.35 | 2-oxoglutarate-dependent dioxygenase |
| **peaxi162scf00024g02219** | 2.72 | -1.57 | 0.82 | 2-oxoglutarate-dependent dioxygenase |
| **peaxi162scf00047g02318** | 2.77 | -5.52 | 2.68 | 2-oxoglutarate-dependent dioxygenase |
| **peaxi162scf00337g00926** | -1.57 | 1.34 | -0.19 | 2-oxoglutarate-dependent dioxygenase |
| **peaxi162scf00540g00218** | 3.16 | -1.16 | 0.42 | 2-oxoglutarate-dependent dioxygenase |
| **peaxi162scf00559g00019** | 2.45 | -3.48 | 3.42 | 2-oxoglutarate-dependent dioxygenase |
| **peaxi162scf00294g00812** | -6.12 | 4.03 | -0.21 | ACO1-like |
| **peaxi162scf00712g00513** | -1.38 | 1.84 | -0.98 | ACO1-like |
| **peaxi162scf00047g01927** | 2.82 | -0.58 | 1.18 | ACO-like |
| **peaxi162scf00141g00330** | -5.24 | 0.36 | 1.51 | ACO-like |
| **peaxi162scf00074g01725** | -6.14 | 2.08 | 3.78 | ACS |
| **peaxi162scf00620g00121** | 3.06 | -0.82 | 0.50 | ACS10 |
| **peaxi162scf00790g00212** | -4.24 | 2.21 | -1.32 | Acyl-CoA N-acyltransferases (NAT) |
| **peaxi162scf00075g01418** | -1.89 | 1.02 | -0.13 | bHLH |
| **peaxi162scf00325g00034** | 2.64 | -2.23 | 1.54 | ETR2 |
| **peaxi162scf00332g00526** | 2.66 | 2.61 | -1.44 | HLS1 |
| **Auxin** |  |  |  |  |
| **peaxi162scf00124g01719** | -0.97 | 2.03 | -1.31 | SAUR-like |
| **peaxi162scf01044g00226** | -1.51 | 2.09 | 0.54 | Auxin induced gene, IAA13 |
| **peaxi162scf00464g00039** | -2.41 | 2.24 | -4.46 | SAUR-like |
| **peaxi162scf00740g00554** | -3.83 | 2.34 | -0.46 | SAUR-like |
| **peaxi162scf00533g00001** | -2.26 | 2.38 | 1.76 | SAUR-like |
| **peaxi162scf00683g00461** | -2.57 | 2.53 | -0.36 | auxin efflux carrier |
| **peaxi162scf00043g02713** | -4.28 | 2.59 | -0.22 | Auxin induced gene, IAA13 |
| **peaxi162scf00334g00610** | -5.73 | 2.71 | -3.67 | SAUR-like |
| **peaxi162scf00362g01042** | -3.23 | 2.94 | -4.58 | SAUR-like |
| **peaxi162scf01133g00016** | -4.41 | 3.05 | -3.42 | Auxin-Induced in Root cultures 12 |
| **peaxi162scf00362g01119** | -1.06 | 3.15 | -3.32 | SAUR-like |
| **peaxi162scf00124g01740** | -3.09 | 3.28 | -1.95 | SAUR-like |
| **peaxi162scf00286g00729** | -5.23 | 3.46 | 1.36 | Auxin-responsive family protein |
| **peaxi162scf00464g00323** | -4.34 | 3.56 | -1.34 | SAUR-like |
| **peaxi162scf00362g01191** | -3.10 | 3.72 | -3.20 | SAUR-like |
| **peaxi162scf00168g01124** | -3.03 | 3.90 | -4.04 | SAUR68 |
| **peaxi162scf01412g00012** | -1.11 | 3.96 | -0.35 | Aluminum induced protein |
| **peaxi162scf00016g02523** | -4.65 | 4.27 | 0.04 | IAA-amido synthases |
| **peaxi162scf00684g00013** | -0.58 | 4.41 | -4.21 | SAUR-like |
| **peaxi162scf00000g43013** | -2.74 | 4.53 | -2.47 | Aluminum induced protein |
| **peaxi162scf00362g01116** | -2.65 | 4.73 | -2.82 | SAUR-like |
| **peaxi162scf00340g00811** | -4.11 | 4.73 | 0.83 | IAA-amido synthase |
| **peaxi162scf00334g10111** | -5.74 | 5.87 | -3.95 | SAUR68 |
| **peaxi162scf00362g01118** | -5.23 | 6.07 | -5.92 | SAUR-like |
| **peaxi162scf00362g01117** | -4.02 | 6.10 | -3.95 | SAUR-like |
| **peaxi162scf00464g00324** | -7.55 | 6.16 | -4.60 | SAUR-like |
| **Others** |  |  |  |  |
| **peaxi162scf00164g00126** | -3.02 | 0.21 | 1.00 | GA feedback 2 (AGF2) |
| **peaxi162scf01514g00021** | 3.40 | -0.33 | 0.12 | GA requiring 3 (GA3) |
| **peaxi162scf00111g00923** | 2.51 | 2.87 | -0.68 | Gibberellin 2-beta-dioxygenase 1 |
| **peaxi162scf00130g01126** | 2.86 | -5.71 | 1.00 | Gibberellin 2-beta-dioxygenase 1 |
| **peaxi162scf00082g02010** | 5.34 | -2.83 | 1.19 | Gibberellin 2-oxidase 8 (GA2OX8) |
| **peaxi162scf00111g00920** | 5.10 | 3.29 | -1.08 | Gibberellin 2-oxidase 8 (GA2OX8) |
| **peaxi162scf00015g00525** | -5.64 | 1.80 | - | Gibberellin 3-oxidase 1 (GA3OX1) |
| **peaxi162scf00449g00013** | -4.06 | 1.21 | - | Gibberellin-regulated family protein |
| **peaxi162scf00017g03268** | 4.54 | -3.23 | 0.57 | bHLH |
| **peaxi162scf00074g00201** | 0.08 | 1.85 | -0.54 | Brassinosteroid-responsive RING-H2 |
| **peaxi162scf00111g01140** | 3.22 | -0.84 | 0.48 | Phyb Activation suppressor 1 (BAS1) |
| **peaxi162scf00166g00212** | 3.44 | -1.65 | 3.51 | Cytochrome P450 85A |
| **peaxi162scf00205g00004** | -6.94 | 2.04 | -1.40 | EXORDIUM like 5 (EXL5) |
| **peaxi162scf00235g00217** | -1.27 | 1.00 | -0.10 | EXORDIUM like 5 (EXL5) |
| **peaxi162scf00305g00815** | 2.92 | -0.96 | 0.17 | CYP72A14 |
| **peaxi162scf00449g00512** | -1.62 | 0.63 | 1.04 | Squalene monooxygenase |
| **peaxi162scf00491g00067** | -2.80 | 2.41 | 0.75 | Phyb Activation suppressor 1 (BAS1) |
| **peaxi162scf00498g00217** | 6.06 | -0.91 | 0.40 | Phyb Activation suppressor 1 (BAS1) |
| **peaxi162scf00515g00517** | 2.59 | -2.13 | 0.94 | CLM |
| **peaxi162scf00546g00536** | -1.35 | 1.14 | -2.02 | Phyb Activation suppressor 1 (BAS1) |
| **peaxi162scf03693g00010** | 3.06 | -0.74 | 0.15 | CYP72A7 |
| **peaxi162scf01010g00014** | -4.96 | 2.21 | -0.74 | UGT85A5 |
| **peaxi162scf00030g01110** | -3.74 | 5.30 | -1.16 | UGT85A2 |
| **peaxi162scf00815g00243** | 2.66 | -4.18 | 3.89 | UGT73C2 |
| **peaxi162scf00611g00215** | -2.31 | 1.23 | 0.69 | UGT85A2 |
| **peaxi162scf00138g00092** | -1.89 | 1.36 | 1.29 | UDP-Glycosyltransferase |
| **peaxi162scf00101g01910** | -1.62 | 1.01 | -1.17 | AtCKX5 |
| **peaxi162scf00110g00028** | 2.58 | 1.52 | -0.33 | UDP-Glycosyltransferase |
| **peaxi162scf00130g00727** | 2.71 | 0.44 | 0.11 | Zeatin O-xylosyltransferase |
| **peaxi162scf00347g00627** | 2.96 | -0.85 | 0.70 | Arabidopsis response regulator (ARR) |
| **peaxi162scf00164g00155** | 3.46 | - | - | UDP-glucosyl transferase |
| **peaxi162scf00498g00427** | 3.60 | -2.61 | 0.42 | UDP-glucosyltransferase 73B2 |
| **peaxi162scf00760g00221** | 3.74 | 0.64 | 0.41 | UDP-glucosyl transferase |
| **peaxi162scf00217g00008** | 3.73 | 0.54 | -0.47 | UGT85A5 |
| **peaxi162scf00160g00002** | 4.20 | -0.95 | 1.43 | UGT73B2 |
| **peaxi162scf00673g00224** | 5.10 | -0.50 | -0.27 | CYTOKININ-INDEPENDENT 1 |
| **peaxi162scf00856g00015** | -0.74 | 4.99 | -2.01 | UGT85A2 |
| **peaxi162scf00017g00161** | 9.92 | -2.07 | 1.41 | UGT73C5 |
| **peaxi162scf00312g00710** | 0.96 | -6.28 | 3.20 | UGT73C2 |
| **peaxi162scf00094g00912** | 1.89 | -3.77 | 2.09 | UDP-Glycosyltransferase |
| **peaxi162scf00217g00412** | 4.23 | -2.42 | 1.49 | Abscisic aldehyde oxidase 3 (AAO3) |
| **peaxi162scf00045g00725** | -5.30 | 3.02 | 1.04 | ABA 8'-hydroxylase |
| **peaxi162scf00681g00332** | -1.35 | 1.63 | 0.62 | ABA-responsive protein-related |
| **peaxi162scf00045g00726** | -0.06 | 1.84 | -0.96 | NCED4 |
| **peaxi162scf00038g02025** | 2.47 | -1.95 | -0.09 | lipoxygenase 3 (LOX3) |
| **peaxi162scf00280g00065** | -2.32 | -1.32 | 1.43 | AOS |
| **peaxi162scf00684g00578** | 9.15 | -3.14 | 3.22 | AOS |
| **peaxi162scf00688g00362** | 3.69 | -2.02 | 1.21 | 12-oxophytodienoic acid reductases |
| **peaxi162scf00895g00019** | 3.01 | -2.67 | 0.03 | Lipoxygenase 2 (LOX2) |
| **peaxi162scf00895g00113** | 3.68 | -3.10 | -0.04 | Lipoxygenase 2 (LOX2) |
| **peaxi162scf48302g00001** | 2.61 | -1.82 | -0.22 | Lipoxygenase 2.3 |
| **peaxi162scf00045g01732** | -0.45 | 1.85 | 0.05 | UGT74F1 |
| **peaxi162scf00047g00116** | -1.33 | 1.57 | -1.67 | Benzoate carboxyl methyltransferase |
| **peaxi162scf00047g01123** | -1.41 | 1.55 | -1.53 | SABATH methyltransferase |
| **peaxi162scf00047g01129** | -1.63 | 1.73 | -1.09 | SABATH methyltransferase |
| **peaxi162scf00265g00033** | 2.61 | -0.45 | 0.24 | Tetratricopeptide repeat (TPR)-like |
| **peaxi162scf00303g00048** | 3.55 | -1.39 | 0.30 | UGT74F1 |
| **peaxi162scf00883g00811** | 5.26 | 0.40 | -0.25 | UGT74E2 |

**Supplementary** Table S3 Unigenes associated with Transcription factors exhibiting a Log2 FC≥1 and p≤0.05 in at least one transition.

| **Gene ID** | **FCD2/D0** | **FCD4/D2** | **FCD7/D4** |
| --- | --- | --- | --- |
| **AP2/EREBP** | | | |
| **peaxi162scf00716g00032** | -5.21 | 1.33 | -1.25 |
| **peaxi162scf00167g00083** | -1.22 | 3.31 | 0.28 |
| **peaxi162scf01094g00002** | 3.12 | -1.18 | 0.13 |
| **peaxi162scf01464g00006** | -3.96 | 2.50 | -0.77 |
| **peaxi162scf00365g00043** | -3.34 | 1.66 | -0.87 |
| **peaxi162scf00301g00122** | -2.52 | 1.47 | -0.52 |
| **peaxi162scf00072g00229** | -2.42 | 1.55 | -0.66 |
| **peaxi162scf00074g00125** | -1.87 | 1.14 | 0.60 |
| **peaxi162scf00380g00041** | -1.66 | 1.50 | -1.31 |
| **peaxi162scf00227g00048** | -1.42 | 1.22 | -0.97 |
| **peaxi162scf00463g10017** | -1.21 | -1.18 | 1.14 |
| **peaxi162scf00377g00087** | 2.73 | -2.34 | -0.53 |
| **peaxi162scf00063g00115** | 2.96 | -1.56 | 0.79 |
| **peaxi162scf00390g10026** | 4.39 | 0.41 | 1.11 |
| **peaxi162scf00079g02119** | 4.75 | -2.97 | 1.44 |
| **peaxi162scf00618g00058** | 5.46 | 0.97 | 1.22 |
| **peaxi162scf00001g00582** | 6.02 | -5.23 | 3.24 |
| **peaxi162scf00047g02318** | 2.77 | -5.52 | 2.68 |
| **peaxi162scf00332g00526** | 2.66 | 2.61 | -1.44 |
| **peaxi162scf00024g01820** | -3.74 | 5.21 | 0.35 |
| **peaxi162scf00294g00812** | -6.12 | 4.03 | -0.21 |
| **peaxi162scf00167g00083** | 3.64 | 3.31 | 0.28 |
| **Aux/IAA** | | | |
| **peaxi162scf00099g00127** | 2.84 | -1.07 | -0.08 |
| **peaxi162scf00123g00214** | -1.07 | 0.74 | -0.47 |
| **peaxi162scf00345g00027** | 3.61 | -1.89 | 1.93 |
| **peaxi162scf00287g00193** | 5.33 | -1.00 | 1.12 |
| **peaxi162scf00015g00237** | -5.11 | 4.16 | -4.36 |
| **peaxi162scf00043g02713** | -4.28 | 2.59 | -0.22 |
| **peaxi162scf00049g02220** | -1.59 | 2.51 | -1.14 |
| **peaxi162scf00282g00515** | -2.12 | 1.31 | -0.42 |
| **peaxi162scf00578g00523** | -1.78 | 1.94 | -1.09 |
| **peaxi162scf00643g00021** | -4.77 | 1.07 | 1.59 |
| **peaxi162scf01044g00226** | -1.51 | 2.09 | 0.54 |
| **peaxi162scf01311g00016** | -3.46 | 2.47 | -1.25 |
| **peaxi162scf01416g00119** | -0.89 | 2.44 | -1.18 |
| **bHLH** | | | |
| **peaxi162scf00008g03526** | -8.34 | 5.27 | -0.03 |
| **Peaxi162Scf00428g01020** | -1.35 | 1.72 | -0.84 |
| **Peaxi162Scf00285g00311** | 2.22 | -0.10 | 0.63 |
| **Peaxi162Scf00712g00029** | 4.31 | -1.66 | 1.00 |
| **peaxi162scf00015g01031** | 1.43 | 3.02 | 0.81 |
| **peaxi162scf00026g00312** | -2.42 | 0.30 | -1.24 |
| **peaxi162scf00049g02223** | 2.82 | -0.73 | 0.03 |
| **peaxi162scf00087g00093** | 4.08 | -1.99 | 1.21 |
| **peaxi162scf00098g00024** | 5.40 | -3.14 | 1.89 |
| **peaxi162scf00129g00321** | -1.03 | 3.04 | -6.32 |
| **peaxi162scf00201g00004** | 2.93 | -1.82 | -0.07 |
| **peaxi162scf00263g01412** | -2.07 | -1.46 | 1.11 |
| **peaxi162scf00274g00834** | 2.49 | -1.07 | -0.04 |
| **peaxi162scf00281g00710** | 3.39 | -1.58 | 1.08 |
| **peaxi162scf00286g00828** | -1.40 | 2.00 | -1.27 |
| **peaxi162scf00377g00029** | -1.09 | 2.35 | -0.78 |
| **peaxi162scf00428g01020** | -1.35 | 1.72 | -0.84 |
| **peaxi162scf00458g00320** | 4.24 | 0.57 | -3.49 |
| **peaxi162scf00735g00516** | -5.61 | 1.80 | 0.42 |
| **peaxi162scf00980g00018** | 3.48 | -1.18 | 0.24 |
| **peaxi162scf01190g00006** | -0.67 | 4.30 | -0.99 |
| **peaxi162scf00020g02019** | 0.85 | 5.87 | -0.21 |
| **peaxi162scf00380g00816** | 5.34 | 0.50 | -0.29 |
| **peaxi162scf00385g00068** | -2.86 | 1.09 | 0.06 |
| **peaxi162scf00403g00716** | 3.77 | -1.63 | 0.16 |
| **Zinc finger CO-like** | | | |
| **peaxi162scf00007g10129** | 4.62 | -1.23 | -0.11 |
| **peaxi162scf00015g00927** | 4.13 | 1.13 | 0.22 |
| **peaxi162scf00126g00624** | 3.64 | -0.63 | 0.35 |
| **peaxi162scf00128g01749** | -2.87 | 0.47 | 3.13 |
| **peaxi162scf00287g00115** | -2.52 | 1.19 | 0.90 |
| **peaxi162scf00459g00510** | 3.04 | 1.31 | 1.17 |
| **peaxi162scf00564g00613** | -2.22 | 1.27 | 0.36 |
| **peaxi162scf00581g00039** | -6.31 | 1.41 | 4.30 |
| **peaxi162scf00942g00122** | -1.45 | 1.48 | -1.05 |
| **peaxi162scf01346g00018** | 1.96 | 2.18 | -0.11 |
| **Zinc finger Dof** | | | |
| **peaxi162scf00013g00084** | -4.46 | 3.08 | -0.37 |
| **peaxi162scf00087g02224** | -2.56 | 0.02 | 1.78 |
| **peaxi162scf00207g01131** | -3.85 | 3.02 | -1.13 |
| **Zinc finger GATA** | | | |
| **peaxi162scf00354g00048** | 6.86 | -4.22 | 2.85 |
| **peaxi162scf00370g00058** | -7.11 | 1.80 | 0.42 |
| **peaxi162scf01175g00018** | -4.02 | 0.21 | 1.63 |
| **peaxi162scf01286g00002** | -1.79 | 1.56 | -1.50 |
| **Zinc finger C2H2** | | | |
| **peaxi162scf00003g00267** | 6.24 | -4.60 | - |
| **peaxi162scf00013g00059** | -2.07 | 2.07 | 0.33 |
| **peaxi162scf00016g00251** | 2.16 | 1.90 | -0.52 |
| **peaxi162scf00016g00288** | -1.63 | 1.17 | -0.97 |
| **peaxi162scf00128g01758** | 3.97 | -0.02 | 0.40 |
| **peaxi162scf00149g01017** | 2.98 | 0.55 | -0.77 |
| **peaxi162scf00149g01022** | 3.02 | 0.10 | -0.28 |
| **peaxi162scf00207g01365** | -4.15 | 0.21 | 3.26 |
| **peaxi162scf00494g00027** | 2.92 | -1.74 | 0.99 |
| **peaxi162scf00526g00069** | 2.85 | -1.43 | 0.77 |
| **peaxi162scf00683g00026** | 2.74 | -1.04 | 0.48 |
| **peaxi162scf00845g00005** | -2.54 | 2.93 | -0.61 |
| **Zinc finger C3H** | | | |
| **peaxi162scf00813g00016** | 2.78 | -1.46 | 0.26 |
| **peaxi162scf00003g10415** | 2.99 | -1.49 | 0.68 |
| **peaxi162scf00032g00917** | 2.72 | -1.27 | 0.32 |
| **peaxi162scf00877g00126** | 4.55 | -2.49 | 2.17 |
| **homeodomain-leucine zipper protein** | | | |
| **peaxi162scf00620g00710** | -1.87 | 1.02 | 0.10 |
| **peaxi162scf00074g00025** | 5.13 | -2.75 | 0.82 |
| **peaxi162scf00189g00919** | -2.14 | 1.64 | -0.19 |
| **peaxi162scf00009g00422** | 3.36 | -0.57 | 1.27 |
| **peaxi162scf00096g01614** | -2.27 | 1.36 | -1.49 |
| **peaxi162scf00767g00212** | 2.85 | -1.80 | -0.66 |
| **peaxi162scf00624g00067** | 2.89 | -0.12 | 0.02 |
| **peaxi162scf00932g00044** | 3.02 | -1.17 | -0.48 |
| **peaxi162scf00007g01020** | 2.17 | 3.38 | - |
| **peaxi162scf00190g01523** | 0.69 | 3.36 | -0.77 |
| **Peaxi162Scf00092g01520** | -5.84 | 7.68 | 0.88 |
| **MADS-box** | | | |
| **peaxi162scf00023g02913** | -2.86 | -1.50 | 2.56 |
| **peaxi162scf00021g00015** | -2.77 | -1.05 | 2.88 |
| **peaxi162scf01178g00019** | 5.69 | -1.18 | 0.59 |
| **peaxi162scf00688g00218** | -2.14 | -0.40 | 2.25 |
| **peaxi162scf00370g00071** | 1.23 | 2.53 | -1.45 |
| **peaxi162scf00017g03246** | -2.86 | -1.50 | 2.56 |
| **MYB** | | | |
| **peaxi162scf00080g00064** | 2.46 | -2.30 | 0.74 |
| **peaxi162scf00002g00037** | -3.56 | 2.24 | -0.42 |
| **peaxi162scf00404g00810** | 2.72 | -0.65 | -1.17 |
| **peaxi162scf00578g00008** | -2.22 | 4.43 | -3.16 |
| **peaxi162scf00578g00007** | -2.09 | 3.65 | -2.88 |
| **peaxi162scf00276g00412** | 4.32 | -1.10 | 0.63 |
| **peaxi162scf01256g00017** | 2.94 | -2.27 | -0.20 |
| **peaxi162scf00385g00066** | 3.84 | -1.61 | -0.58 |
| **peaxi162scf00391g00621** | -2.79 | 1.66 | -0.53 |
| **peaxi162scf00268g00046** | -1.79 | 1.38 | -0.71 |
| **peaxi162scf00033g00251** | -1.50 | 1.60 | -0.56 |
| **peaxi162scf00986g00227** | 6.61 | -1.74 | 0.92 |
| **peaxi162scf00797g00013** | -3.21 | 1.61 | 0.29 |
| **peaxi162scf00452g00412** | -4.32 | 3.71 | 2.47 |
| **peaxi162scf00132g01412** | -1.74 | 3.67 | -0.87 |
| **peaxi162scf00170g01336** | 2.03 | -1.20 | 3.07 |
| **peaxi162scf00294g00813** | 3.09 | -4.03 | 3.62 |
| **peaxi162scf00020g01234** | 3.70 | -0.79 | 0.82 |
| **peaxi162scf00092g01519** | 7.22 | -1.83 | -0.06 |
| **peaxi162scf01096g00316** | 8.83 | -4.21 | 1.39 |
| **peaxi162scf00207g01353** | 1.58 | -2.74 | 1.26 |
| **WRKY** | | | |
| **peaxi162scf00121g00018** | -3.00 | 1.52 | 0.33 |
| **peaxi162scf00691g00417** | 6.10 | -0.55 | 0.87 |
| **peaxi162scf00073g02335** | -3.79 | 1.51 | 1.19 |
| **peaxi162scf00843g00220** | -1.39 | 1.03 | 0.92 |
| **peaxi162scf00055g01910** | -3.46 | -0.41 | 1.65 |
| **peaxi162scf00459g00841** | -5.04 | 2.11 | 1.26 |
| **peaxi162scf00007g00315** | -1.12 | 1.59 | 0.71 |
| **peaxi162scf00469g00624** | -3.74 | 3.67 | 2.33 |
| **peaxi162scf00401g00087** | 2.71 | -1.40 | 0.80 |
| **peaxi162scf00585g00513** | 0.68 | 2.38 | -1.58 |
| **HSF** | | | |
| **peaxi162scf00002g02718** | 5.52 | -5.46 | -0.32 |
| **peaxi162scf00259g00710** | 5.80 | -1.74 | 0.85 |
| **peaxi162scf00301g00033** | 2.57 | -0.73 | 0.39 |
| **peaxi162scf00327g00413** | 2.87 | -1.24 | 0.28 |
| **peaxi162scf00433g00411** | -4.85 | 3.27 | -0.06 |
| **peaxi162scf00516g00645** | 2.75 | -1.12 | 0.21 |
| **peaxi162scf00546g00118** | -1.77 | -0.57 | 2.30 |
| **peaxi162scf00915g00029** | 2.67 | -0.15 | 0.47 |
